# Effects of Late-Life Health Transitions on Spouses’ Psychological Well-Being

**DOI:** 10.1093/geroni/igab046.1149

**Published:** 2021-12-17

**Authors:** Lynn Martire, Ruixue Zhaoyang, Christina Marini

**Affiliations:** 1 The Pennsylvania State University, University Park, Pennsylvania, United States; 2 Adelphi University, Garden City, New York, United States

## Abstract

Declining physical health likely affects not only older adults’ own well-being, but also that of their spouse. Using two waves of data from 610 couples in the National Social Life, Health and Aging Project, we examined effects of health declines over five years on change in self and spousal psychological well-being. Actor-Partner Interdependence Model findings showed that declines in spouses’ physical health (i.e., increased pain and decreased physical and cognitive function) predicted increases in older adults’ anxiety. Given the increasing importance of later-life social ties outside of marriage, we further considered the role of non-spousal health confidants. Preliminary findings suggest that effects of health declines on both partners’ well-being depend on the availability of these confidants. When older adults have people in addition to their spouse with whom they can talk about their health, detrimental effects of spouses’ declining health on older adults’ well-being are weakened for some health outcomes

